# They promised this ten years ago. Effects of diabetes news characteristics on patients’ perceptions and attitudes towards medical innovations and therapy adherence

**DOI:** 10.1371/journal.pone.0255587

**Published:** 2021-08-19

**Authors:** Hans Vehof, Eibert R. Heerdink, José Sanders, Enny Das

**Affiliations:** 1 Centre for Language Studies, Radboud University, Nijmegen, The Netherlands; 2 Research Group Process Innovations in Pharmaceutical Care, HU University of Applied Sciences, Utrecht, The Netherlands; 3 Division Pharmacoepidemiology & Clinical Pharmacology, Utrecht Institute for Pharmaceutical Sciences, Utrecht, The Netherlands; International Medical University, MALAYSIA

## Abstract

Patients have ever-increasing access to web-based news about hopeful scientific developments that may or may not cure them in the future. Science communication experts agree that the quality of news provision is not always guaranteed. However, literature does not clarify in what way users are actually affected by typical news characteristics such as the news object (described developmental phase of an innovation), the news source (degree of authority), and the news style (degree of language intensification). An online vignette experiment (N = 259) investigated causal relationships between characteristics of news about diabetes innovations and patients’ perceptions of future success, their interest in the innovation, and attitudes regarding current therapy adherence. Findings show that descriptions of success in mice led to higher estimations of future success chances than earlier and later developmental phases. Furthermore, news from a nonauthoritative source led to an increased interest in the innovation, and a more negative attitude towards current lifestyle advice. Lastly, the intensification of the language used in news messages showed slight adverse effects on the readers’ attitude. These findings, combined with their small effect sizes, support the optimistic view that diabetes patients are generally critical assessors of health news and that future research on this topic should focus on affected fragile subgroups.

## Introduction

In the present digital era, patients with a chronic condition frequently encounter news messages about the potential healing of their disease. No matter if the person is an active health news seeker, or perhaps is trying to avoid such information, exposure to some extent seems likely for many. A large number of digital platforms actively spread scientific research results, with varying goals [1, 2]. For example, web-based newspapers present readers with quality interviews with scientists or patient organizations [3] acting as an information conduit for their communities; social media platforms provide members with daily news updates often from a highly personal perspective; academic and governmental news platforms and scientific libraries often the actual primary source of new scientific results. This online news on scientific health developments differs widely in important news characteristics such as news objects (i.e. fragsments of contenst that receive a focus of the editor, such as the scientific *developmental* phase of an innovation in the news), the news source (with a degree of *authority*), and the news style (e.g. degree of *language intensification*). Given the unrestrained expansion of web-based news sources, essential questions arise that yet require empirical answering in the scientific literature: *In what way do typical characteristics of news about innovative treatments affect patient perceptions*? The present study aims to obtain new insights into this topic in the context of a chronic disease that receives a lot of media attention worldwide: diabetes mellitus.

### Diabetes research in the news

Diabetes mellitus is one of the most widely spread chronic diseases and it receives much attention in academic papers as well as in newspapers and social media [4–6]. A quick scan in scientific databases such as PubMed shows that, annually, tens of thousands of academic papers are published about both diabetes mellitus type 1 and type 2, from which authors of health news could potentially draw. Although significant progress towards a cure for diabetes is reported in several publications, various promising innovative concepts have been covered for decades without clinically adaptable results. Already in 1972, for example, a glucose sensor was developed that purportedly gave rise to optimism about a so-called *’self-contained totally implantable artificial organ that would continuously monitor sugar concentration in a body fluid of a diabetic and meter out insulin in proportion to need’* [7]. The whole organ is presently referred to as the artificial pancreas and is still under development. News media regularly report early stages of medical research with innovative potential while remaining unclear about actual clinical evidence from patients and thus about market potential. In fact, a study showed that less than 1 out of 5 Dutch newspaper articles reporting innovative treatments for diabetes were supported by an in-article reference to proven effects in actual patients [8].

Nonetheless, even the holy grail in research, the randomized controlled trial on actual patients, may lack reliability and still reach media headlines. Weaker human trials have small sample sizes or include patients with narrow inclusion criteria. It may also happen that they detect evidence for effectivity, despite the fact that risk-benefit ratios of the treatment are unfavorable due to high costs or side effects. Fortunately, several examples of media watchdogs have arisen in the world to assess the quality of health news articles and to educate news editors and the general public on interpreting the value of health claims [9, 10].

### News object: Developmental phases in medical science

Developing new drugs or other medical therapies is a complicated and time-consuming process. In addition to the lengthy process of discovering new molecules or refining smart techniques and testing their effectiveness in laboratory settings, the subsequent clinical research phase, involving experiments on smaller and larger groups of actual patients, can last years and is not always successful. For example, innovations in the endocrine disease area that are promising enough to be tested for safety on humans (i.e., phase 1 clinical research) have a chance of about 14% to be eventually approved for the market, which is between 6 and 9 years later [11]. The development of new medical applications can take decades and follows distinct phases, from first ideas to actual market access. In the earliest stages of research, evidence from observational studies (e.g., correlations with food) or evidence from fundamental research may lead to animal-testing, clinical testing in small groups of humans, or encourage investments in innovative technologies. Randomized clinical trials in patient populations provide the most valuable type of evidence: proof of effectiveness and a probability that the new therapy will be applicable in large populations in the future largely increased.

The academic literature shows no indication that choices regarding the dissemination of diabetes news are affected by the research phase of the presented innovation. Innovations in all stages of research are discussed in news media. Although lay readers might not be able to differentiate between evidence from different clinical phases, such messages may still affect treatment perceptions and emotions. This assumption is supported by research findings showing that the preclinical phase (e.g., research on animals) elicited the most positive emotions among diabetes patients on Facebook [12]. Persons with knowledge of clinical research may presumably perceive a qualification like ’successful in patients’ as news with higher actual success chances. However, overly optimistic perceptions might potentially harm treatment adherence, Literature by Mann et al. [13] showed that disease and medication beliefs that were inconsistent with a chronic disease model of diabetes were significant predictors of poor medication adherence.

### News source: Authority of the news source

The accessibility of the internet and the low cost to spread information has led to increased access to and dissemination of health information. Patients seeking health information encounter large quantities of information from various sources and of varying quality and accuracy. The new digital era entails a significant challenge in assessing the credibility of health news [14]; and this is also true for diabetes news [15]. Particularly relevant is the source of health information since important health-related behaviors and decisions are based on the perceived credibility of the source [16]. On the internet, perceived credibility is determined by the authority of the administrator of a website or platform, representative of the expertise, and the trustworthiness of its writers of health information [17, 18].

The primary source of a health message frequently is a research institute or academic press release. Nonprofessional websites without authority repeatedly are selecting sources of health information [19]. Most health news messages reach the public through a broad range of selecting sources, varying from governmental health institutes to information gatekeepers such as news anchors, reporters, and journalists who select and present health news messages for their public. In the online context, technological interfaces such as web-based search engines and social media like Facebook function as selective sources, filtering and forwarding health news messages of primary sources [20].

Interestingly, online networks also enable receivers of health information to select and transfer health news messages themselves (whether or not assisted by technological interfaces). Examples of such receiving sources are moderators and members of online support groups, Facebook groups, chat rooms, and discussion forums. Online users of health news information may establish the selecting sources’ credibility based on the perceived degree of expertise in these sources. In Western societies, credibility will be estimated higher in expert-based, authorized sources such as government health institutes, and lower in nonprofessional sources such as Facebook groups. In line with the Elaboration Likelihood Model of Persuasion [21], it is expected that sources with a clear scientific origin–such as universities or governmental institutions–will, by rule of thumb, lead to higher perceptions of a message’s credibility and accuracy. News messages coming from authoritative sources may therefore increase estimations that the innovation will eventually be successful and personally useful. Further, it can be hypothesized that the authority of the source plays a crucial role in opinion formation and change especially to those (patients) who are less inclined to seek news on the topic and thus are less likely to take the time to elaborate on the actual arguments in the message through the central route.

### News style: Language intensification

Health news regularly contains powerful language that is used by the author to increase its vividness and attention value. Although this so-called language intensification is a complex concept [22], a functional definition is the use of language that is used to deviate from neutrality [23]; in the present case, that is deviating towards the positive aspect. Strong words such as breakthrough, enormous, very important, or lifesaving in a sentence intensify, by inserting pars pro toto’ superlatives’, a statement that otherwise would be more factual; this phenomenon is not strange to coverage of medical news [24] and may affect the reader. Although overall results are mixed, several studies show that the use of language intensifiers increases the clarity of the message [25, 26]. In another study, language intensification led to high message elaboration by receivers: after reading intensified language, patients were better able to distinguish stronger from weaker arguments [27]. In experimental studies, the high-intensive language had a more positive influence on attitudes and intentions than low-intensive language [28]. Also, some studies suggest that language intensification may influence behavior [29].

In contrast, a study on perceptions of health news messages showed that these were mostly affected by objective risk characteristics; language intensification only affected readers’ perceptions of the severity of a health risk [30]. These findings suggest that readers of health news have the ability to correct for language intensification and see the objective part of the information. Although the use of language intensification is common practice in health news coverage, specific effects of adding strong words to health news, on outcomes (i.e., attitudes, and behavioral intentions) among patient populations are yet unknown. The present study compared intensified with factual text versions, to assess effects on the value that diabetes patients attach to the news message content, and thus on their estimations that the presented innovation will be successful. Furthermore, the study assessed effects on interest in the given innovation and attitudes towards current lifestyle advice and therapy adherence attitudes and intentions.

Despite excessive media attention for clinically unproven innovations and existing worries about this phenomenon among experts [8, 31, 32], the actual effects of such media coverage on patients have not been established in empirical research. It is known that characteristics of news messages are associated with emotions [12, 33, 34], but empirical evidence on patients’ attitudes and behaviors to continue a challenging lifestyle program or medication regimen is lacking. The current study aims to gather first insights into the possible impact of reading about promising medical research, varying the typical characteristics of scientific health news. Focus is put on dependent outcomes that represent perceptions and attitudes regarding the presented innovation in the news, and regarding the current treatment that the patients receive. The outcomes may be considered determinants of actual therapy adherence behavior [35].

To measure news effects on these outcomes, we present fictional innovations in short messages that are systematically manipulated on three news dimensions; object, source, and style, that is: (a) the *developmental phase* of a particular innovation; (b) the type of *authority* of the source on the innovation news message; and (c) the degree of language intensification by in-text presence of strong words to emphasize the innovative research results.

Based on the literature and the guidelines outlined above, the research questions that guided the current research were as follows:

#### Research question

When patients diagnosed with diabetes read news messages about innovative ways to treat their disease in the future: to what extent do news message characteristics (i.e. developmental phase of the innovation, authority of the news source, and language intensification) affect patients’ (i) expectations of the innovation’s success, (ii) interest to gain additional information, and intentions towards currently prescribed (iii) lifestyle advices and (iv) medication regimens?

## Materials and methods

### Participants and design

Human participants were involved in an online vignette experiment. The local review board *UPPER (Utrecht University)* approved our study protocol (September 28th, 2018; UPF1806) and declared that the study did not fall under the scope of the “Medical Research Involving Human Subjects Act”. Prior to start of the actual digital survey, patients were informed about the aims and burden of the study and consent was given by a *click to accept* principle. Because, no names and other identifying information was requested, a signed consent form was not required.

Diabetes patients for the present online vignette study were recruited both on social media and in pharmacies. Patients were invited by posting a link on three Dutch diabetes-related forums and 14 diabetes Facebook groups (October 2018 –January 2019). With the approval of the website owners or moderators, a recruitment text with a request to participate in an online questionnaire about Diabetes News and Reader’s Mood was posted together with the survey link. In the same period, 25 Dutch pharmacies agreed to contribute to our research by handing out flyers containing a recruitment text and a weblink to customers treated for diabetes mellitus type 1 or 2. All participants were volunteers and remained anonymous.

To include sufficient patients in our experiment, we determined a clinically significant increase of interest in an innovation of 0.5 on a 5 point Likert scale (with a standard deviation for the population of 1.0). A sample size calculation for an exploratory study with alpha 0.05 and power 0.80 predicted a necessary sample size of 63 per experimental group. To detect differences between two authority types, two language intensity types or three developmental phases, we needed a total of 126 to 189 participants.

The present experiment consisted of a three-factor between-subjects design, using vignettes, i.e., short descriptions of imaginary situations. Participants were randomly assigned to one vignette that varied on three different dimensions: (1) three levels of the developmental phase that the presented innovation could be in, (2) two types of authority of the source, and (3) two degrees of language intensification (see Measures section). This design resulted in a total of twelve (3x2x2) unique text combinations and the messages were matched on diabetes type (type 1 and 2). Although a within-subjects design may result in more power to detect differences, it would come at the potential costs of a lower external validity and a higher dropout rate. Moreover, the present research anticipated that reading the second vignette in a short period of time may have led to decreased emotional responses.

### Materials and procedure

Our data was collected in Lime Survey and was exported to a protected University network environment. No data was saved that can lead to tracing individual participants of the study.

**Fig 1:**
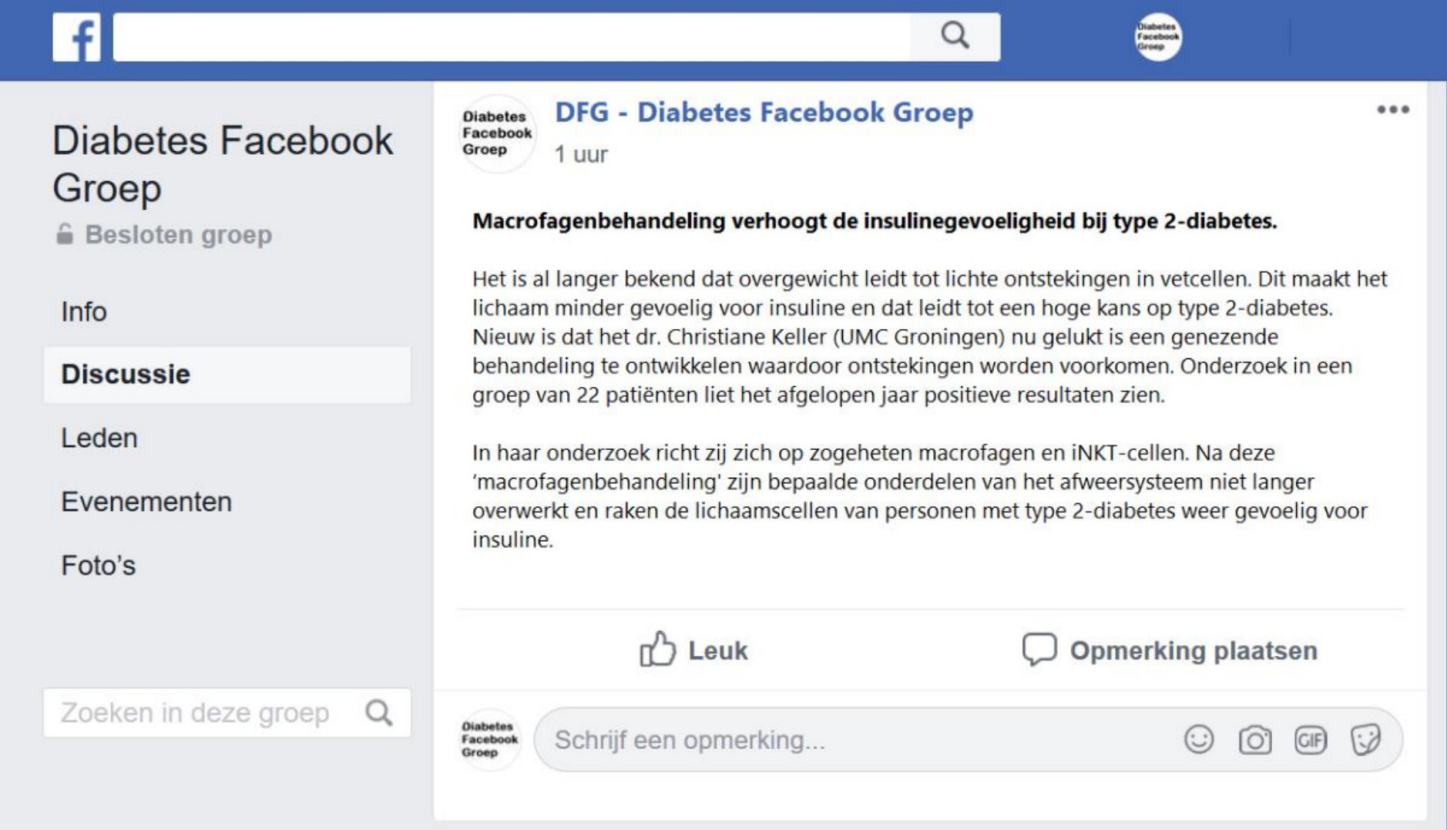
The visual frame surrounding the news message suggests that the news message is published in an online diabetes Facebook group.

**Fig 2:**
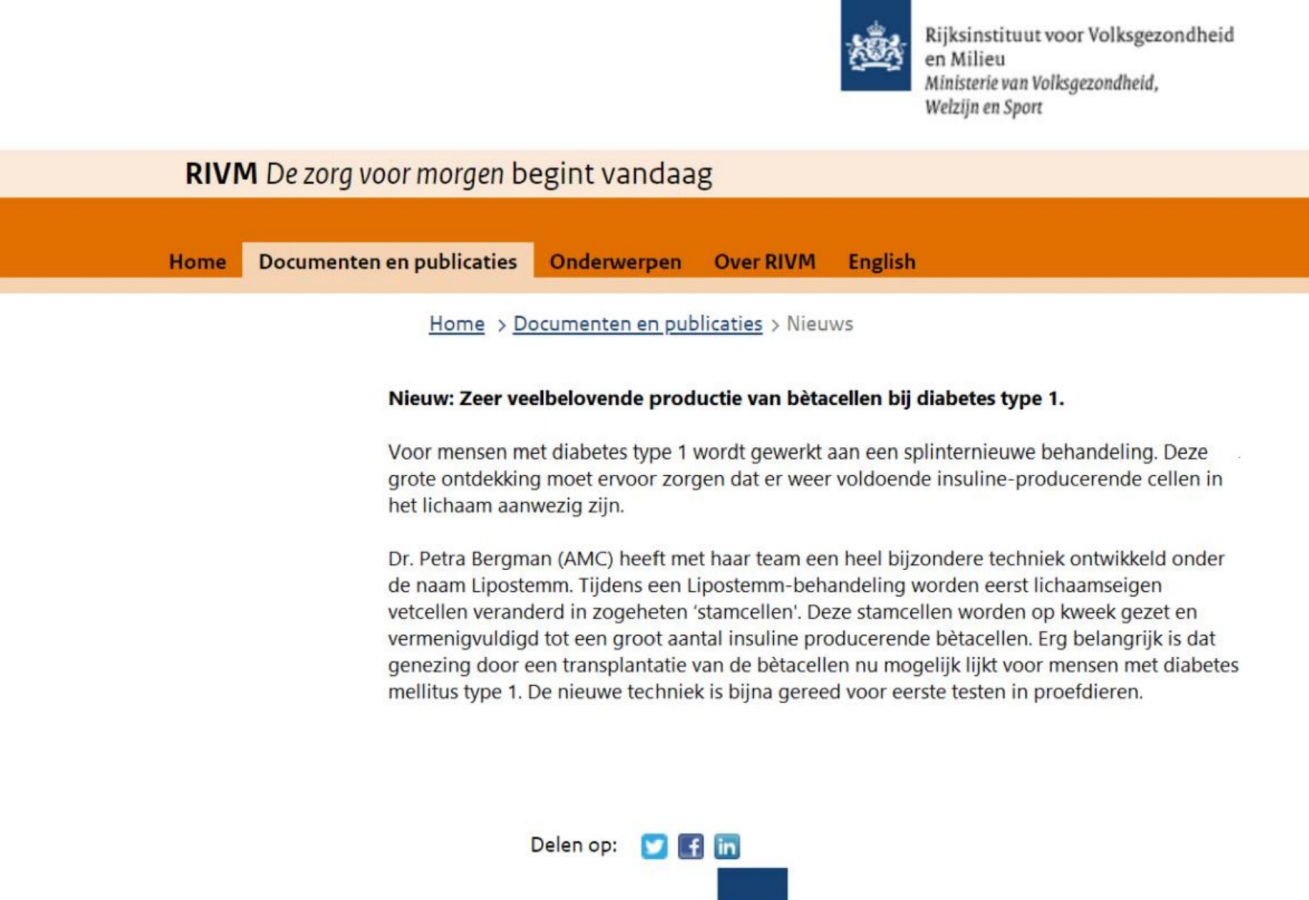
The visual frame surrounding the news message suggests that the news message is published on the website of an authority: Netherlands National Institute for Public Health and the Environment (RIVM).

After giving consent for participating, all participants indicated their age and whether they were diagnosed with either type 1 or type 2 diabetes mellitus. Those not diagnosed with diabetes, or having an age under 18 years, were thanked for their interest, and the questionnaire automatically ended. There were no other in- or exclusion criteria. The remaining participants were then asked to read one of 12 experimental news texts that were shown in visual frames (Figs 1 and 2). The survey software matched the fictional news messages to typical innovations for either diabetes mellitus type 1 or type 2. Thus, the messages were matched to specific diabetes mellitus type, but this matching was not a factor (or: manipulation) in the vignette research design.

To experimentally manipulate the presented scientific evidence for the innovation’s success, we varied the developmental phases in the vignettes. Table 1 shows the three simplified stages of diabetes research (levels of presented evidence) that we used. For the fundamental phase, we presented the following statement: the innovation is soon ready to be tested in animals. Next, to indicate that preclinical evidence (in animals) had already been found, we stated that the therapy showed positive results in mice. Lastly, to indicate that actual clinical evidence in patients was gathered, we stated that the treatment proved successful in 22 patients.

**Table 1:** Factors and levels used in the construction of the vignettes.

Factor	Level
Developmental phase	Reported success in fundamental research stages: ’*the therapy is soon ready to be tested in mice’*
	Reported success in preclinical research on mice: *’In the last year*, *the therapy showed positive results in mice’*
	Reported success in clinical research on patients: ’In the last year, *the therapy showed positive results in a group of 22 patients’*
Source authority	Source with authority: simulated website of the Dutch National Institute for Public Health and Environment (RIVM)
	Source without authority: a not further specified ’*Diabetes Facebook Group’* simulation
Language intensification	No intensified news content; base texts
	Intensified news content; four common intensifiers added (*special*, *discovery*, *important*, *and promising*)

To suggest that the message was either visible on the website of (1) an authoritative governmental source (i.e., Dutch National Institute for Public Health and Environment), or (2) from a nonprofessional non-authoritative source (i.e., a Facebook group named Diabetes Facebook Group without specified author).

To experimentally manipulate language intensification, we added four language intensifiers (special, discovery, important, and promising) that are frequently used in Dutch medical news to 50% of the presented vignettes, and this was added in combination with degree indicators very, a lot, much, and many; all of these intensifiers were absent from the other 50%. The four chosen intensifiers were identified after programmatically counting all words and identifying potential text intensifiers that were written in 173 web-based news articles about diabetes that we selected for earlier research.

After reading the experimental message, participants filled out a web-based baseline questionnaire that assessed four dependent outcomes (see Measures section): (1) perception of successfulness of the innovation; (2) interest in the innovation; (3) attitude towards advised lifestyle changes; (4) attitude towards adhering to medication regimes.

### Measures

Factor analysis was conducted for 8 items measuring (1) estimations of future successfulness and (2) interest in gaining additional information 3) intentions regarding the usefulness of previously received lifestyle advices (4) intentions regarding adhering to current medication regimens. A principal component analysis was used to generate the factors. The Kaiser-Meyer-Olkin (KMO) test was used for determining the sample adequacy. A value of more than 0.5 has been considered adequate to perform factor analysis [36]. The Bartlett test of Sphericity was used to determine the homogeneity of the data. A Bartlett test p-value of less than 0.05 is considered significant and useful for factor analysis [36]. Oblimin rotation was used after the initial factor solution. The optimal number of factors was assessed from the scree plot. Findings showed that the following four 2-item (5-point Likert) scales represented the four components of our interest:

1. Two items assessed the patient’s expectations of the innovation’s success on a 5-point Likert scale (strongly disagree–strongly agree): "The treatment I just read about will be successfully used against diabetes in 10 years", and "The message I just read exaggerates the success of the new treatment (reverse coded)" (Cronbach’s alpha = 0.69). The average score on this 2 item scale was used to answer research questions.
2. Two items assessed the patient’s interest to gain additional information on a 5-point Likert scale (strongly disagree–strongly agree): "I will look up more information about this new treatment", and "I will discuss this treatment with my health professional" (Cronbach’s alpha = 0.83). The average score on this 2 item scale was used to answer research questions.
3. Two items assessed the patient’s intentions regarding received lifestyle advice: "It is wise to follow lifestyle advice in the coming month", "I will follow the lifestyle advice that I received in the coming month", (strongly disagree–strongly agree) (Cronbach’s alpha = 0.80). The average score on this 2-item scale was used to answer research questions.
4. Two items assessed the patient’s intentions regarding current medication adherence: "It is wise to take prescription medication in the coming month" I will take my own medication as prescribed in the coming month" (strongly disagree–strongly agree) (Cronbach’s alpha = 0.91). The average score on this 2-item scale was used to answer research questions.

### Statistical analysis

Data were analyzed with SPSS Version 25.0 (SPSS, Inc, IL, USA) using unifactorial analyses of variance with developmental phase, source platform, language intensification as independent factors, and 3x2x2 ANOVA to assess interactions between independent variables. Effect sizes were calculated with partial eta squared, with effect sizes of .01–.06 considered as small, .06–.14 as medium and above .14 as large [37]. Descriptive analysis was carried out using mean and standard deviation with the range for continuous variables, while frequency and percentages were used for discontinuous ones. The present study did not use corrections for multiple testing based on the exploratory nature of the research, with research questions but without a prespecified key hypothesis. In exploratory studies multiple test adjustments are not strictly required [38]. Moreover, corrections such as the Bonferroni correction may come at the cost of missing a novel point of departure for studying new associations between independents and clinical outcomes [39].

This study was part of a larger research project that aims to assess the effects of health news characteristics on patient well-being; associations between patient characteristics and health seeking preference will be reported elsewhere. To secure the outcomes of a random vignette distribution, and possibly correct for unequal patient characteristics in the various vignette groups, we were able to verify the equal distribution of different baseline characteristics: age, gender, the estimated number of years since diabetes diagnosis, and current medication.

## Results

We collected data on independent and dependent variables from 259 participants. Table 2 shows the participant characteristics. Table 3 shows the random allocation to the vignette manipulations. since a small number of the participating patients did not complete the full questionnaire, the total number of patients in the various analyses shows a small variation between 235 and 259 (see results section). Sensitivity analyses with these patients showed no relevant differences in outcome between the subgroups (data available upon request).

**Table 2:** Characteristics of the participants.

	*N* = 259	Range
Age, mean (SD)	50.6 (15.5)	18–80
Gender, *n* (%)		
Female	176 (68.0)	
Male	78 (30.1)	
Other	5 (1.9)	
Diabetes Type, *n* (%)		
T1DM	110 (42.5)	
T2DM	149 (57.5)	
Education, *n* (%)		
High^1^	107 (41.3)	
Middle^2^	106 (40.9)	
Low^3^	46 (17.8)	
Years since diagnosis, mean (SD)	14.4 (12.1)	<1–57
Medication use, *n(%)*		
Insulin + other medication	63 (24.3)	
Insulin only	106 (40.9)	
Other medication only	71 (27.4)	
No medication	19 (7.3)	

^1^ Bachelor, Master.

^2^ Senior general secondary education (HAVO), university preparatory education (VWO), vocational education and training (MBO 2,3,4)^.^

^3^ Primary school, preparatory vocational secondary education (VMBO), Vocational education and training (MBO1).

**Table 3:** Distribution of vignette manipulations.

	N = 259
Developmental phase, n (%)	
Reported success in fundamental stage	88 (34.0)
Reported success in mice	84 (32.4)
Reported success in 22 patients	87 (33.6)
Language intensification, n (%)	
Intensified	138 (53.3)
Not intensified	121 (46.7)
Source platform, n (%)	
Expert	132 (51.0)
Laymen	127 (49.0)

### Developmental phase

The ANOVA on patients’ *expectations of the innovation’s success*, entering the manipulation of developmental phase as a fixed factor, showed a difference between the manipulated phases with a small effect size (*F*(2, 258) = 3.81, *p* < .05, η_p_^2^ = .029). The highest success chances were perceived when success in mice was reported (*M* = 1.93, *SD* = 0.42). See Table 4.

**Table 4:** Results one-way ANOVA: between group differences as filled in on a 5 point likert scale 0–4; mean/neutral = 2.0 (Developmental phase).

	Developmental phase, expressed by Success			
	1: ’Soon ready for research on mice’ Mean(*SD*)	2: Success in mice’ Mean(*SD*)	3: Success in 22 patients’ Mean(*SD*)	*F* statistic
				P value
				η_p_^2^ =
Expectations of the innovation’s success	1.74(0.44)	1.93(0.42)	1.78(0.52)	*F*(2,258) = 3.81
	n = 88	n = 84	n = 87	*p* = .023*
				η_p_^2^ = .029
Interest to gain additional information	1.72(1.17)	1.70(1.18)	1.40(1.04)	*F* (2,256) = 2.25
	n = 87	n = 83	n = 87	*p* = .11
				η_p_^2^ **=** .017
Lifestyle intention	3.05(0.89)	3.03(0.74)	2.84(0.97)	*F* (2,234) = 1.42
	n = 83	n = 75	n = 77	*p* = .25
				η_p_^2^ = .012
Medication adherence intention	3.40(1.07)	3.21(1.05)	3.46(0.75)	*F* (2,236) = 1.38
	n = 82	n = 74	n = 81	*p* = .25
				η_p_^2^ = .012

No statistically significant main effects of the developmental phase were found on the other dependent variables: interest in the presented innovation, and intentions regarding current lifestyle and medication therapy adherence.

### Source authority

The ANOVA on patients’ *information intention*, entering the manipulation of developmental phase as a fixed factor showed that patients’ information intention was higher when the source was not authoritative, (*F*(1, 256 = 4.79 *p* < .05, η_p_^2^ = .018). In contrast, patients’ medication adherence intention was higher when the source condition was authoritative (F(1, 236) = 8.52, p < .005, η_p_^2^ = .035). A similar pattern was observed for lifestyle intentions, but this effect was not statistically significant. See Table 5 for Means and SDs.

**Table 5:** Results one-way ANOVA: between group differences as filled in on a 5 point likert scale 0–4; mean/neutral = 2.0 (Source authority).

	Source authority		
	Authority	No authority	*F* statistic
			P value
			η_p_^2^ =
Expectations of the innovation’s success	1.81(0.50)	1.83(0.42)	*F* (1,258) = 0.12
	n = 132	n = 127	*p* = .73
			η_p_^2^ = .000
Interest to gain additional information	1.45(1.09)	1.76(1.16)	*F* (1,256) = 4.79
	n = 131	n = 126	*p* < .029*
			η_p_^2^ = .018
Lifestyle intention	3.08(0.83)	2.87(0.90)	*F* (1,234) = 3.24
	n = 117	n = 118	*p* = .073
			η_p_^2^ = .014
Medication adherence intention	3.54(0.77)*	3.18(1.11*)	*F* (1,236) = 8.52
	n = 118	n = 119	*p* = .004*
			η_p_^2^ = .035

### Language intensification

The ANOVA on patients’ *expectations of the innovation’s success*, entering the manipulation of language intensification as a fixed factor, showed no significant effect. Patients’ *information intention* was higher in the no intensification condition, compared with the intensification condition (*F*(1,255) = 3.93, *p* < .05, η_p_^2^ = .015), with a small effect size. Furthermore, patients had a higher *lifestyle intention* in the condition without language intensification, *F* (1,234) = 5.74, *p* < .05, η_p_^2^ = .014. No significant effect was observed on the *medication adherence intention*. See Table 6.

**Table 6:** Results one-way ANOVA: between group differences as filled in on a 5 point likert scale 0–4; mean/neutral = 2.0 (Language intensification).

	Language intensification		
	No intensification	Intensification	*F* statistic
			P value
			η_p_^2^ =
Expectations of the innovation’s success	1.83(0.48)	1.80(0.45)	*F* (1,258) = .34
	n = 121	n = 138	*p* = .56
			η_p_^2^ = .001
Interest to gain additional information	1.75(1.21)	1.47(1.06)	*F* (1,256) = 3.93
	n = 120	n = 137	*p* = .048*
			η_p_^2^ = .015
Lifestyle intention	3.12(0.85)	2.85(0.87)	*F* (1,234) = 5.74
	n = 112	n = 123	*p* = .026*
			η_p_^2^ = .024
Medication adherence intention	3.29(1.07)	3.42(0.87)	*F* (1,236) = 1.03
	n = 111	n = 126	*p* = .31
			η_p_^2^ = .004

### Interaction effects

To assess interactions between the independent variables, 3 (developmental phase) x 2(source authority) x 2(language intensification) ANOVAs were conducted on the dependent variables. The analyses revealed no statistically significant (*p* < .05) interaction effects.

## Discussion

Previous studies reported an abundance of accessible online health messages, frequently lacking clarity regarding their therapeutic potential [8, 40, 41]. The present study assessed whether such news characteristics affected diabetes patients’ expectations, interests, and attitudes regarding the presented innovation and their current therapies. For the first time, a particular focus was put on the persuasive effects of the reported developmental phase of the innovation on perceptions and intentions. In biomedical research on innovative therapies, the evidence for clinical effects in actual patients is vital, and earlier successes must be celebrated with restraint. Only when therapeutic results are confirmed in actual patients, so-called proof of concept is reached, which indicates that in this stage, innovations have improved chances to succeed [11]. However, journalists frequently report about earlier developmental phases, which may affect patients’ perceptions and adherence intentions different from situations where better evidence is available.

This study found that, on average, patients estimate the future success chances of a presented diabetes innovation the highest when success in mice is shown and not when success in patients is presented. Note that the power of the effects was limited and, remarkably, that patients’ interest in gaining additional information did not increase by stating success in actual patients. Apparently, patients are already quite convinced by the value of laboratory medicine, unaware of the challenging translation from such inventions to healthcare innovations. [42] In other words, it may be that patients did not carefully interpret essential signals about the level of scientific evidence due to a lack of medical and healthcare knowledge. This may have induced peripheral argument processing, following the so-called *expert heuristic* ("these scientists can be trusted") rather than following the central route of full argument processing [43]. Especially in experimental settings such as our study, such an expert heuristic may eliminate developmental phase-related persuasive effects that require more cognitive elaboration. In addition, for laymen (such as our participants), the reference to ‘mice’ in the laboratory evidence may function as a rather prototypical cue that the evidence is indeed scientific evidence, and this ‘mice’ reference may thus in fact have reinforced, rather than diminished, the aforementioned expert heuristic.

Additional support for the explanation that at least some patients may have processed information via the peripheral route, comes from the effects of the manipulating factor *source authority*: results showed that when the news was brought by an authoritative professional source, it increased patients’ intention to adhere to current medication regimens, in comparison to reading the same message when brought by a non-authoritative, nonprofessional source. The improved adherence intentions in the authoritative source version may be explained by the persuasive power that authorities such as the *National Institute for Public Health and the Environment* (RIVM) in the Netherlands have. The positive cues that participants received from recognizing this authority may have led to simple merit conclusions and quick information processing in the peripheral route [43]. However, our study also showed that specifically the nonprofessional, non-authoritative source increased patients’ interest in the innovation, which seems not directly in line with this theory. One possible explanation may be that the interest is in fact a need to receive additional information which results from a lower trust in non-authoritative message sources: information brought by such sources is in need of further elaboration and verification.

Remarkably, our findings suggest that intensifying medical news with strong language such as *promising*, *very*, or *breakthrough*, is counterproductive when trying to serve a patient community. Although effect sizes were small, they indicate that using intensified language decreases rather than increases patients’ interest in the presented innovation and even slightly lower patients’ intentions to follow current lifestyle advice. This unexpected, reversed, effect of language intensification in our study may be explained when taking into account that intensified language in the context of our study may have been perceived as an expression of subjectivity. Linguistically, intensification foregrounds the involvement, or *stance*, of the author and as such, it contributes to the emotive and subjective dimension of discourse [44, 45]. Subjective intensification may therefore be perceived–either consciously or subconsciously—as framing bias, in which the author or speaker takes a particular position on arguable topics such as anticipating a better future [46]. In a context in which patients are expectant of trustworthy, factual descriptions of future treatment options, this non-neutral position of the author may thus weaken the persuasiveness of the news message. Note that in the context of news stories on research, language intensification must be distinguished from spin, which was found to increase readers’ estimates of future success of the presented therapy [47]. However, spin, i.e. the conscious or unconscious misrepresentation of study results that overemphasize the efficacy or safety of the treatment; in the current study, was not included in the current study.

### Limitations and future directions

The current study has some limitations. First, anonymous patients were recruited online, and it was not possible to verify their self-reported medical information. Second, a large part of the online recruitment was performed in Facebook groups. This may have led to bias regarding Facebook-related perceptions and opinions since these patients are more used to this social media platform than the whole diabetes population may be. Possibly, participating patients that were recruited from Facebook put more trust in non-authoritative sources while holding a relative distrust towards official authorities. Yet, most of the cooperating Facebook Groups were either linked to large diabetes patient associations that do not oppose science nor medical authorities in any way, or were acting as a conduit for scientific news from universities and other scientific authorities. Moreover, it is this specific population that is most frequently online and reading news on Facebook groups, and can, therefore, be considered as our primary target population. Another limitation may be the absence of a control group that did not read the news at all. The implication of not including this third group is that questions with respect to reading versus not reading news, remain unanswered. However, the odds are low that in a real-world situation, patients will never read health news messages. The last limitation may be the use of parametric tests on data from 5 point Likert scales. Although it has become common practice to perform parametric tests on 5-point scales, some researchers oppose the idea that differences between *neutral*, *agree*, *and strongly agree* are equal and linear steps [48].

Future research should aim at effects on emotional wellbeing as well as on determining specific vulnerable patient groups that are more susceptible to effects than our general population on average was. This can preferably be done by using a longitudinal observational design containing actual media exposure measures on patients from susceptible groups. Furthermore, it may be important to understand if, and how, patients are affected by news on social media accounts of professionals in the medical fields, specifically physicians and scientists. Another limitation is the power of our study. The number of patients that we included was large enough to detect 0.5 point differences on the 5p Likert scales. However, for detecting such a difference in two-way interactions (e.g. source authority x developmental phase) the eventual number of participants was about fifty percent too low. This is a possible explanation for the absence of interaction effects. Hence, we recommend future studies to repeat our experiments with higher power since clinical relevant differences may still exists in diabetes groups. Lastly, research needs to be performed on patients’ understanding and perceptions of medical developments and on possible education: what do patients comprehend, and how much knowledge of current diabetes research is necessary to optimize information provision and quality of life.

## Conclusions

The present experimental study presented diabetes patients with news about relevant medical innovations, to assess whether specific news characteristics, i.e., the developmental phase of an particular innovation; the authority of the source; and the language intensification degree of the message, affect patients’ perceptions of the presented innovation and their current therapy adherence intentions. Large effects were not found. A small but significant effect that was established is an increase of the intention to adhere to medication after reading news from an authoritative source. Though limited, this effect is may yet be of importance, given the large and greatly varied diabetes population that encounters an ever increasing amount of online health news. From an overarching viewpoint, the results of our study support an optimistic view that patients diagnosed with diabetes, as they are generally critical assessors of health news. Future research on this topic should above all focus on affected fragile subgroups.

## Supporting information

S1 Table List with the names of the 14 participating Facebook Groups. (DOCX)
