# Supplementary material for: They promised this ten years ago. Effects of diabetes news characteristics on patients’ perceptions and attitudes towards medical innovations and therapy adherence
Source: PLoS One. 2021 Aug 19;16(8):e0255587. doi: 10.1371/journal.pone.0255587 (PMC8376088; doi:10.1371/journal.pone.0255587)
Supplement: S1 Table — (DOCX) [file pone.0255587.s001.docx]

**S1 Table. List with the names of the 14 participating Facebook Groups.**

| Name | Translation to English |
| --- | --- |
| Gezond afvallen | Loosing weight healthy |
| Het geheim achter diabetes | The secret behind diabetes |
| Koolhydraatarm eten | Eating low carb-style |
| JDRF vrijwilligers | JDRF volunteers |
| Stop diabetes programma | Stop diabetes program |
| Nederlandse obesitas kliniek Beverwijk | Dutch obesitas clinic Beverwijk |
| Strijden tegen obesitas | Combatting obesitas |
| Kinderen en volwassen diabetes type 1 | Children and adults diabetes type 1 |
| Samen staan we sterk | Together we are strong |
| Jongvolwassen en diabetes | Young adults and diabetes |
| Diabetes Fonds | Diabetes foundation |
| Diabetes Vereniging Nederland | Diabetes association Netherlands |
| Diabetes en Zo | Diabetes and so |
| Diabetes vrienden | Diabetes friends |
